# Rural exodus and land use change in northern **M**orocco: 2000–2020

**DOI:** 10.1007/s12517-022-10341-w

**Published:** 2022-06-08

**Authors:** Jesús Gabriel Moreno-Navarro, Kamal Targuisti El Khalifi, Jesús Ventura Fernández, Ismail Hilal, Eduardo López Magán

**Affiliations:** 1 Department of Physical Geography and Regional Geographic Analysis, University of Seville, Seville, Spain; 2 Faculty of Geology, Abdelmalek Essaadi University, Tétouan, Morocco; 3 Sciences and Techniques, National Centre for Nuclear Energy (CNESTEN), Rabat, Morocco

**Keywords:** Rural exodus, Land use change, Morocco

## Abstract

The implementation of the Urban Development Master Plan, SDAU (Schema Directeur d’Amenagement Urbain) in the Tangier-Tetouan region has marked a radical change in the territorial organization of these provinces: this process that began in 2004 with the construction of the port of Tanger-Med, and the infrastructures to support the port and related industrial areas. This action has meant the change of administrative boundaries as the project progressed. Although the demographic dynamics throughout the Kingdom of Morocco is a process of rural exodus, this migratory pattern is more intense in northern Morocco. The Andalusian Geographical Studies group of the University of Seville, together with Abdelmalek Essaadi University, has investigated the impact of such planning on spatial planning in a framework of international university cooperation since 2002. This article will update previous research based on remote sensing techniques and demographic statistics that analyze the changes that occurred over the last two decades. These changes consist of more than a massive displacement of the rural population toward the large cities and coastal towns. It also means the construction of large infrastructures, holiday houses, and a new pattern of settlement in rural areas. This article aims to describe these changes, as well as the trends in the immediate future, using statistical analysis and the evolution of land uses with GIS and remote sensing tools.

## Introduction

### Mobility and demography in Morocco

The Strait of Gibraltar is one of the most unequal borders in the world, as far as the human development index and per capita income are concerned (Moreno-Navarro and Moreno-Navarro 2011). However, this gap is gradually closing in recent years, with a positive change in the north of this African kingdom. In relative terms, it must be taken into account that the per capita income of the Tangier-Tetouan provinces has traditionally been lower than those of the Casablanca-Settat and Rabat-Salé-Kénitra regions (Haut Commissariat au Plan, HCP 2021), which occupy the highest concentration of industrial production of the Kingdom (Sedra and El Bayed 2021). This gap has been reduced since the implementation of the SDAU (Moreno-Navarro and Moreno-Navarro 2011).

The axis of this master plan is the construction (2004) and commissioning (2007) of the port of Tangier Med which is a strategic project for the Moroccan government. It has involved large investments in infrastructures that have also attracted real state developments. Massive construction activity has also attracted local labor from both rural and coastal areas. This pressure, therefore, has been both on the social plane, spurring the rural exodus, and on the environment, too, with the appearance of quarries, urban growth, abandonment of farming, forest fires, and transport of aggregate. Population growth and housing construction are a constant throughout the kingdom, as can be seen in Tables 1, 2, 3, and 4. Both indicators have been much more evident in the area of the former provinces of Tangier and Tetouan. This growth brings with the rural exodus and the concentration of population in large cities, as well as an increase in second homes building that have been seduced by the official establishment of the royal summer residence on the coast of M’diq.

**Table 1:** Urban population growth (prepared by the authors based on HCP ( 2020))

	1994	2004	2014	Growth
Tangier-Tetouan	928.027	1.197.071	1.647.088	77,5%
Morocco	13.429.658	16.463.634	20.432.439	57,1%

**Table 2:** Rural population growth (prepared by the authors based on HCP)

	1994	2004	2014	Growth
Tangier-Tetouan	162.487	224.000	178.784	9,8%
Morocco	12.644.059	13.428.074	13.415.803	6,1%

**Table 3:** Urban housing growth (prepared by the authors based on HCP ( 2020))

	1994	2004	2014	Growth
Tanngier-Tetouan	928.027	1.197.071	1.647.088	135,7%
Morocco	3.115.583	3.566.667	4.807.743	90,6%

**Table 4:** Rural housing growth (prepared by the authors based on HCP)

	1994	2004	2014	Growth
Tangier-Tetouan	33.588	37.334	36.063	−3,4%
Morocco	1.920.829	2.225.509	2.506.063	30,5%

The two main cities, Tangier and Tetouan, as well as the neighboring municipalities, have accounted for most of the growth. This is a case of urban macrocephaly, which is typical in developing countries (Faraji et al. 2016).

According to World Bank estimates, in the 1960s, Morocco’s population growth was more likely to accelerate than to stabilize, and shortly thereafter, demographers warned this trend would jeopardize development efforts (Sabagh 1993). However, this growth trend would project a population of 37 million for the year 2010, when the reality is that this figure had not yet been reached in 2014 (HCP, 2014). This fact indicates a moderation in growth with birth control and a drastic decrease in mortality and an increase in life expectancy, rising from 64 in 1990 to 75 years in 2015, and an average age that grows from 19.8 to 27, 9 years in the same interval (United Nations Development Program 2019). It must also be considered the internal migration dynamics with a movement toward the northern regions, favored by international immigration with the aim to reach Europe; a 10% of the Moroccan population resides outside their country, and approximately 7% of its annual Gross Domestic Product (GDP) comes from remittances (World Bank 2010). Since the 1990s, in response to new labor opportunities in southern Europe, spatial diffusion of international out-migration has occurred beyond the historical migration belts to regions that used to be predominantly oriented toward internal migration, and one of these belts is Larache and south of Tangier (De Haas 2009).

Migration in Morocco is also attracted by the dynamism of the northern regions of the country because of the process of urban and infrastructure development. In this case, the mobility model would correspond to Zelinsky’s second stage of the mobility transition mode, with the qualification of “early society in transition” with rural exodus, movements toward areas of greater relative development, as well as national and international migrations (Zelinsky 1971). This state occurs precisely at the beginning of a demographic transition like the one Morocco has experienced for the last 30 years. In the present case, transit and economic migrants originating from sub-Saharan Africa and living in big cities or in rural parts of Morocco must be considered (Üstübici, 2015).

The evolution of these population movements and changes in land use have been analyzed with statistical data on population and housing, as well as remote sensing and GIS techniques.

### Objectives

This article aims to describe the patterns that have ruled rural migration and urban development as landscape-modifying processes. Since 2014, we do not have updated statistical data on the population, but the movements are evident on the ground. These movements have their mark on land use, so remote sensing techniques are suitable to describe the trends in land occupation and its relationship with rural exodus. The results can provide arguments for an improvement in the actions for land planning in Northern Morocco, where the lack of control and protocols for the implementation of the legislative framework on urban planning and protection of the environment leads to negative effects on environmental protection. The results of this work are intended to join the current scientific production concerned about control and resilience in the face of such an intense and rapid process.

## Sources and methodology

Statistical sources from the Moroccan Department of Statistics (HCP 2020) about population and housing in the municipalities have been used to quantify the evolution of the census. The Landsat imagery from the United States Geological Survey (USGS) has been processed to produce land cover cartography, and a vector layer from a PAIDAR-MED cooperation project containing municipality limits (Ministry of State, et al. 1996) has also been used to georeference both statistical and satellite imagery data sources. Population censuses in Morocco take place in years ending in number 4, so population data is only available for 1994, 2004, and 2014. Statistical monitoring of these data was complicated due to changes in administrative boundaries, with the appearance and disappearance of municipalities as well as the appearance of new prefectures and provinces. The initial limits of the provinces of Tangier and Tetouan have been maintained for the spatial analysis, as they contain the entire study area. These changes in the administrative limits have been considered in a GIS, updating the attributes of the municipalities layer with population data from HCP.

As a consequence of the management needs already foreseen in the territory, the strategic actions framed in the SDAU have caused changes in the administrative division of the two northern provinces, Tangier and Tetouan, appearing the new provinces of Fahs-Anjra and M’diq-Fnidq. These provinces have later seen their limits rectified, too, as a result of the SDAU revision.

Land use changes have been assessed with imagery from Landsat 5 and Landsat 8 platforms (USGS, 2020). The selection of these platforms has been fundamentally due to their availability and affordability. Landsat 5 platform has operated beyond the expected operational life due to the inconveniences suffered by its successors, Landsat 6 and Landsat 7. We subsequently used the Landsat 8 imagery data with greater radiometric precision, but the same spatial resolution (30 m). A supervised classification (maximum likelihood) and land use cluster with reflectance data of the images corresponding to the months of August of the years 2000, 2014, and 2020 has been carried out. To obtain the training camps, several expeditions were conducted throughout the region with the support of cooperation funds from the Spanish International Cooperation for Development Agency (AECID), Abdelmalek Essaadi University, and the University of Seville. In these expeditions, GPS markings were taken to geocode land uses. These marks would later be used for the preparation of the training camps and signature files. All the processes of classification and mapping have used ESRI ArcGis 10.4. The timing of these field works, together with the quality and availability of Landsat images, have been the reasons for choosing the image acquisition dates. We consider the year 2000 as the starting situation, 2014 as the year with the maximum activity and expansion in construction, and 2020 as the last year of analysis. The latter would be considered as a stage of maturity in the transformation process. The selection has also taken into account the same season and the quality of the images. Images from 2004 have been discarded, as they contain visible effects from the SDAU on the ground.

Landsat images that have been used for the final land use mapping:

Bands that have been used from the platform Landsat-5 Thematic Mapper ™ are as follows:

- Band 1 Visible (0.45–0.52 μm) 30 m
- Band 2 Visible (0.52–0.60 μm) 30 m
- Band 3 Visible (0.63–0.69 μm) 30 m
- Band 4 Near-infrared (0.76–0.90 μm) 30 m
- Band 5 Near-infrared (1.55–1.75 μm) 30 m

Date 22-8-2000

Bands that have been used from the platform Landsat-8 Operational Land Imager (OLI) are as follows:

- Band 1 Visible (0.43–0.45 μm) 30 m
- Band 2 Visible (0.450–0.51 μm) 30 m
- Band 3 Visible (0.53–0.59 μm) 30 m
- Band 4 Red (0.64–0.67 μm) 30 m
- Band 5 Near-infrared (0.85–0.88 μm) 30 m

Dates 21-8-2014 and 21-8-2020

Although other dates and bands have been used, the selection of the images and their final composition have been made according to the clarity of the results. The land uses have been grouped according to the interest of the research, with the following classes: vigorous vegetation, urban and bare soil, mixed farming, and inland waters. For the type of study that we carry out in Morocco, a differentiation between urban land and bare land based on remote sensing techniques is not feasible since a much more detailed study would be needed, implying a different scale of work. One of the problems with the differentiation between both uses would be the segmentation of houses, roads, green areas, etc. It would be necessary to make an exhaustive classification of the cover classes and forms in small portions of land, requiring an effort (Herold et al. 2003) (Barnsley et al. 1993) to differentiate such close spectral signatures exceeding the goals of this work. Likewise, citing the aforementioned about the classifications of the cover classes and forms, it is necessary to have a cartography of the urban surfaces and adjoining lands. The diversity on the surface requires working with different spatial, geometric, spectral, and temporal resolutions (Qihao 2012). It must also be taken into account that this type of technique is not applied to an extension of the size of the Tangier Tetuan region. All this would also depend on the quality of planning that has been implemented in terms of the physical landscape, city growth, and urban expansion. In certain cases, the urban phenomenon can produce a homogeneous signature, as occurs in some areas of Sub-Saharan Africa (Balogun et al. 2011). But in the case of the North of Morocco, we find a predominantly Mediterranean climate with dry summer (Csa) (Köppen 1931) and recent urban planning that, in many cases, alternates green and sports areas between buildings. The tiles of the Landsat images cover an area of 900 m^2^ where a great diversity of smaller surfaces may coincide and whose classification does not fit the goals set out above. It is for these reasons that the studies that have made an effort to differentiate bare and urban land cover focus on smaller areas than the region that is the object of this study. On the other hand, the extractive activities related to the construction of housing and infrastructures produce eventual semi-arid landscapes whose existence is related to this urban growth itself.

**Fig. 1:**
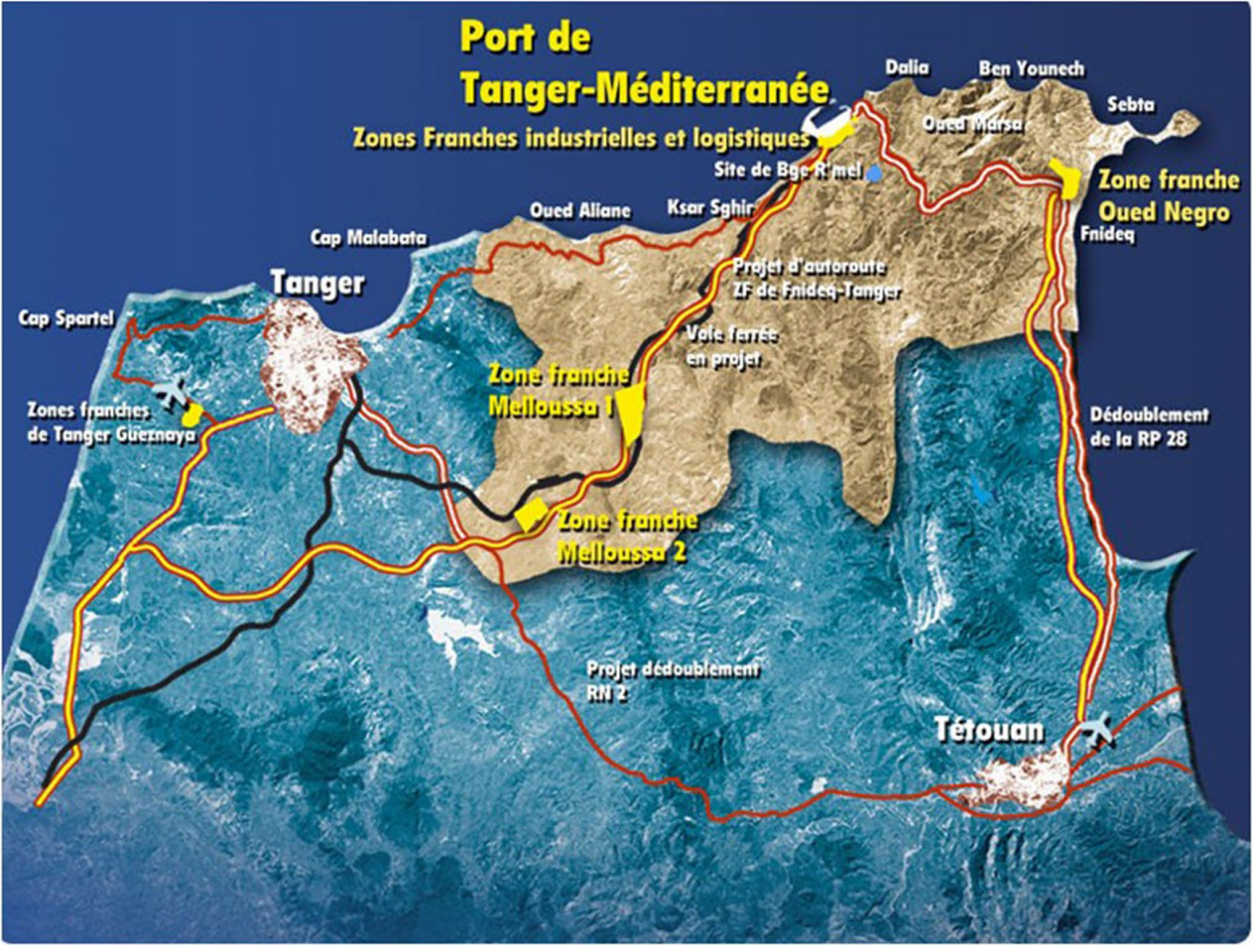
Map of the SDAU. Tangier Méditerranée Special Agency (TMSA) (Navarro and Fernández 2008)

Although the map extent initially covers both provinces, the focus is finally on the new province of Mdiq (Fig. 1, number 4) since it is where more anthropic pressure has been detected. It so happens that the speed of transformation has exceeded the legislative capacity on urban planning in this new province, where more anthropic pressure has been detected (Nejjari 2021)

Statistical and land use results have been verified on the ground and with Google Earth images on an urban growth area (Marina Smir, Mdi'q-Fnideq) and another in rural areas (Halka Zaitoune, Tetouan) which acquisition dates – when available – are close to Landsat selected imagery and with a proper quality of visualization, absence of clouds fundamentally. In this case, the Google Earth images were taken in during the summer of 2006, 2014, and 2020, following the sequence of (a) initial situation, (b) maximum activity, and (c) maturity.

## Results

It has been found that there is negative growth in neighboring municipalities with areas of urban expansion on the coast and in areas far away from the peri-urban areas of the cities of Tangier and Tetouan (Fig. 3). The disparity between population growth and housing growth is different in the Tangier and Tetouan settings (Fig. 3 and Fig. 4). On the coastline near Tetouan, growth in habitat and resident population is uneven. In both cases, housing growth prior to population growth can be seen, but in Mdi’q and Tetouan, this has not been the case, with construction growing faster than the resident population. The analysis of land uses has resulted in a decrease in natural vegetation and agricultural activities between 2000 and 2014, coinciding with the years of the greatest construction activity, both for real state and civil infrastructures. The progression of urban and bare land has followed an inverse evolution, increasing in the years of the greatest activity to end up decreasing in 2020 (Tables 5 and 6).

**Table 5:** Land use changes in the area: percentage (prepared by the authors based on USGS data)

%	2000	2014	2020
Inner water	1	1	1
Vigorous veg.	34	24	26
Mixed farming	43	28	40
Urban and bare soil	22	48	33

**Table 6:** Changes in the P. of M’diq: percentage (prepared by the authors based on USGS data)

%	2000	2014	2020
Inner water	1	1	1
Vigororus veg	47	33	27
Mixed farming	39	16	25
Urban and bare soil	13	50	47

**Fig. 2:**
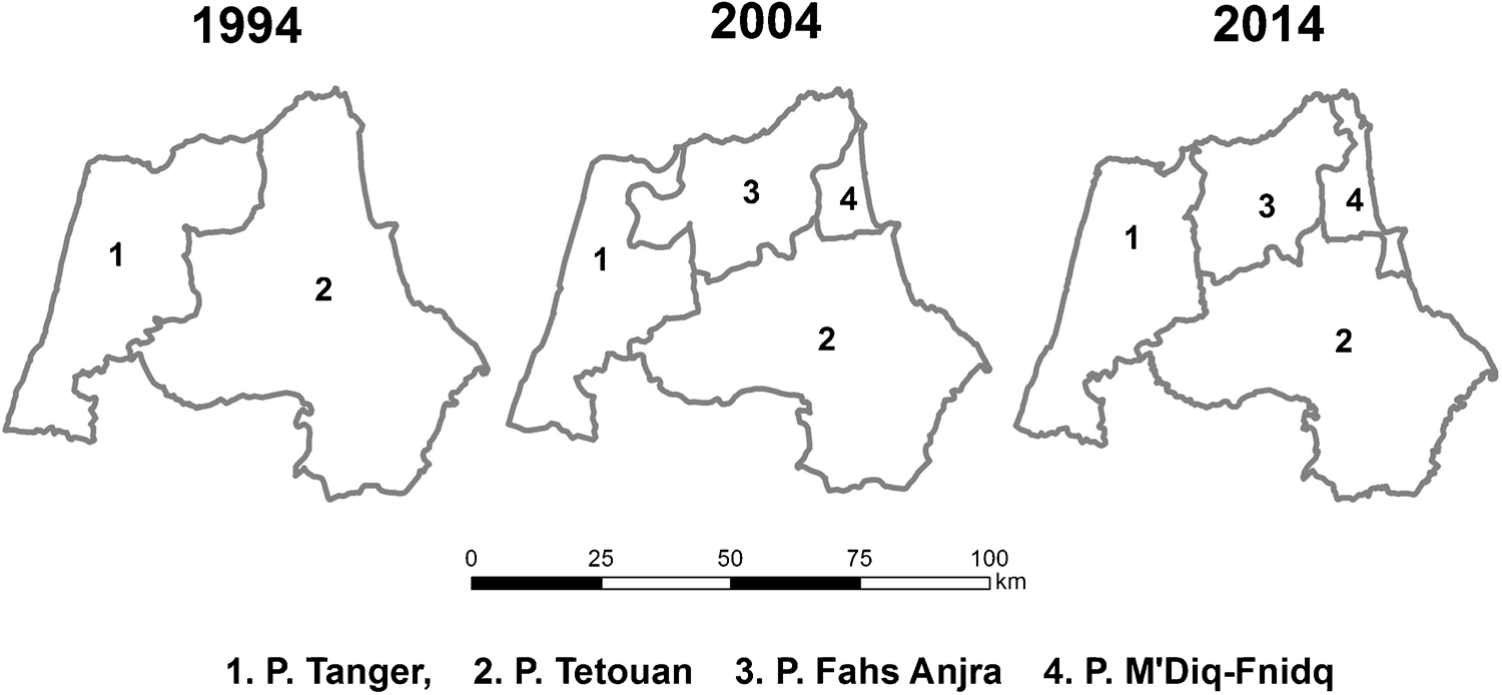
Changes in administrative division (prepared by the authors based on HCP (2020), BORM ( 2003), and PAIDAR-MED)

**Fig. 3:**
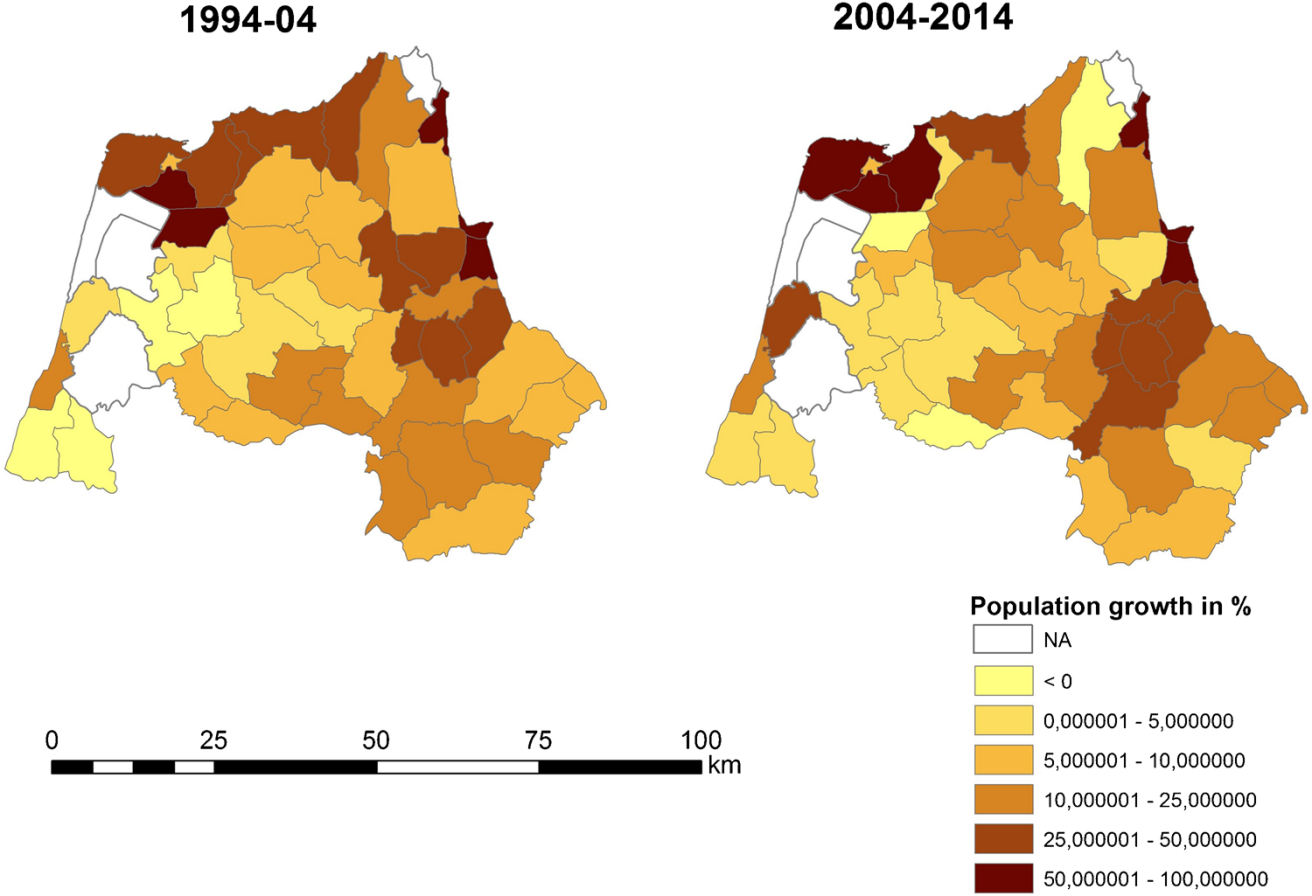
Population growth in percentage (prepared by the authors based on HCP ( 2020))

**Fig. 4:**
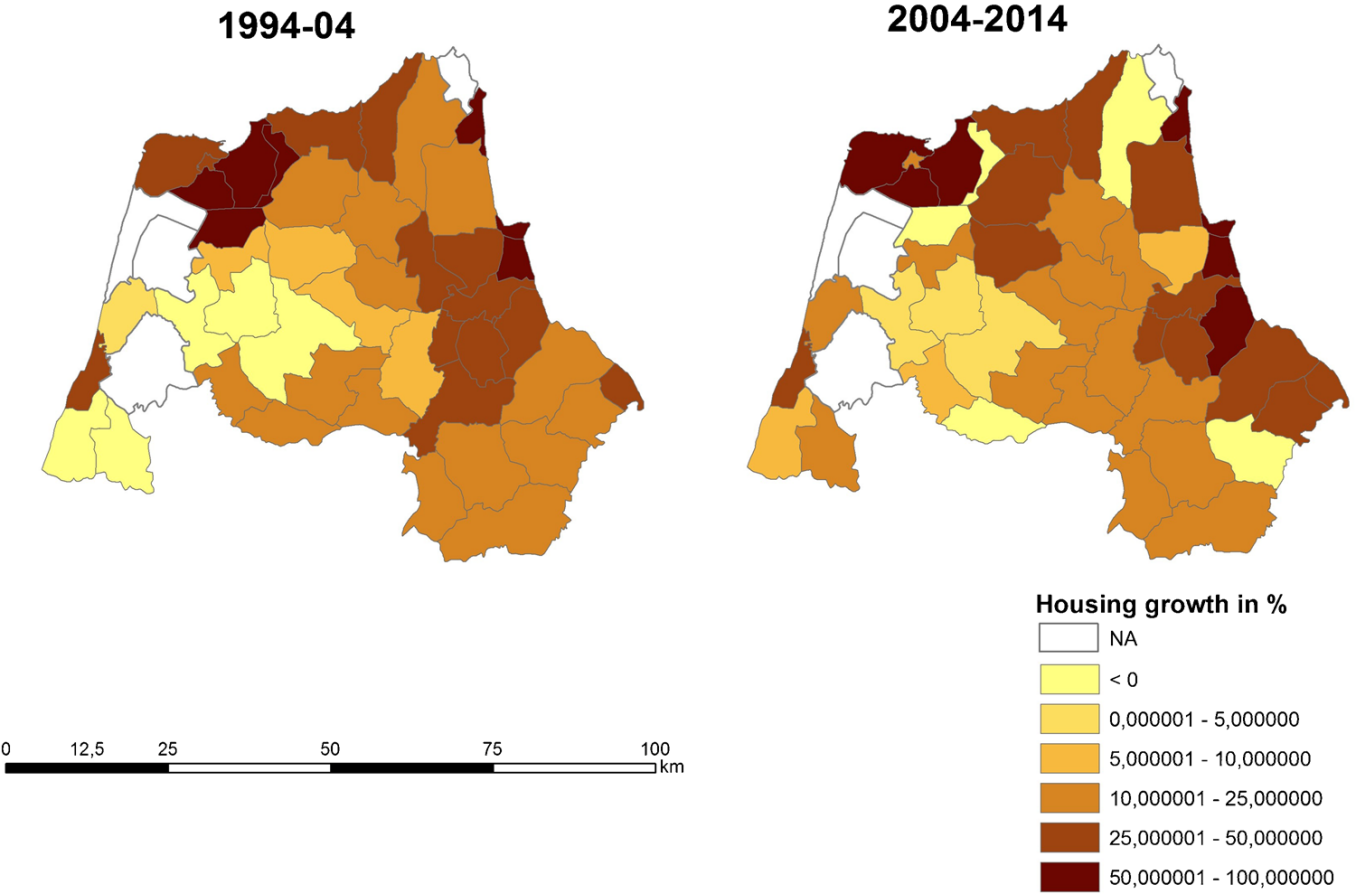
Housing growth in percentage (prepared by the authors based on HCP ( 2020))

**Fig. 5:**
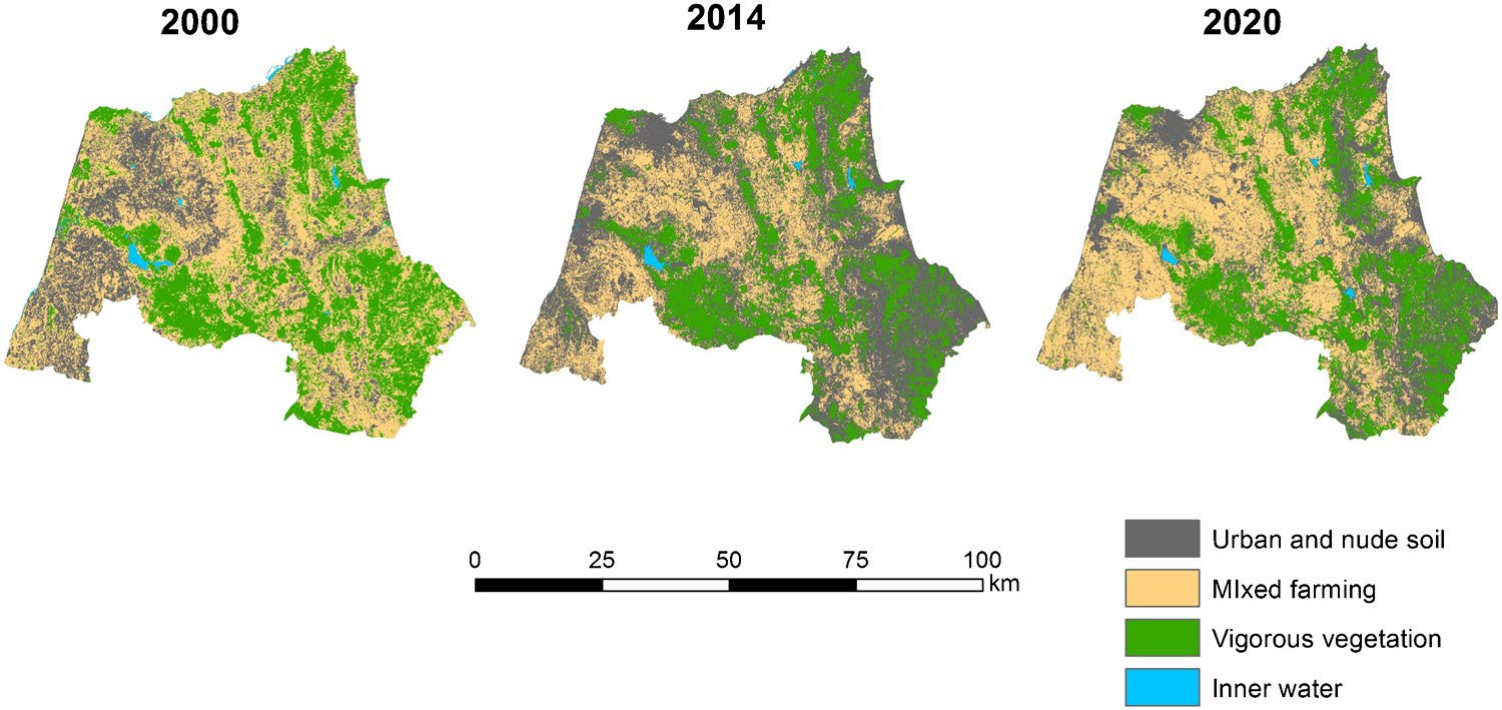
Land use classification in Tangier-Tetouan (prepared by the authors based on USGS)

**Fig. 6:**
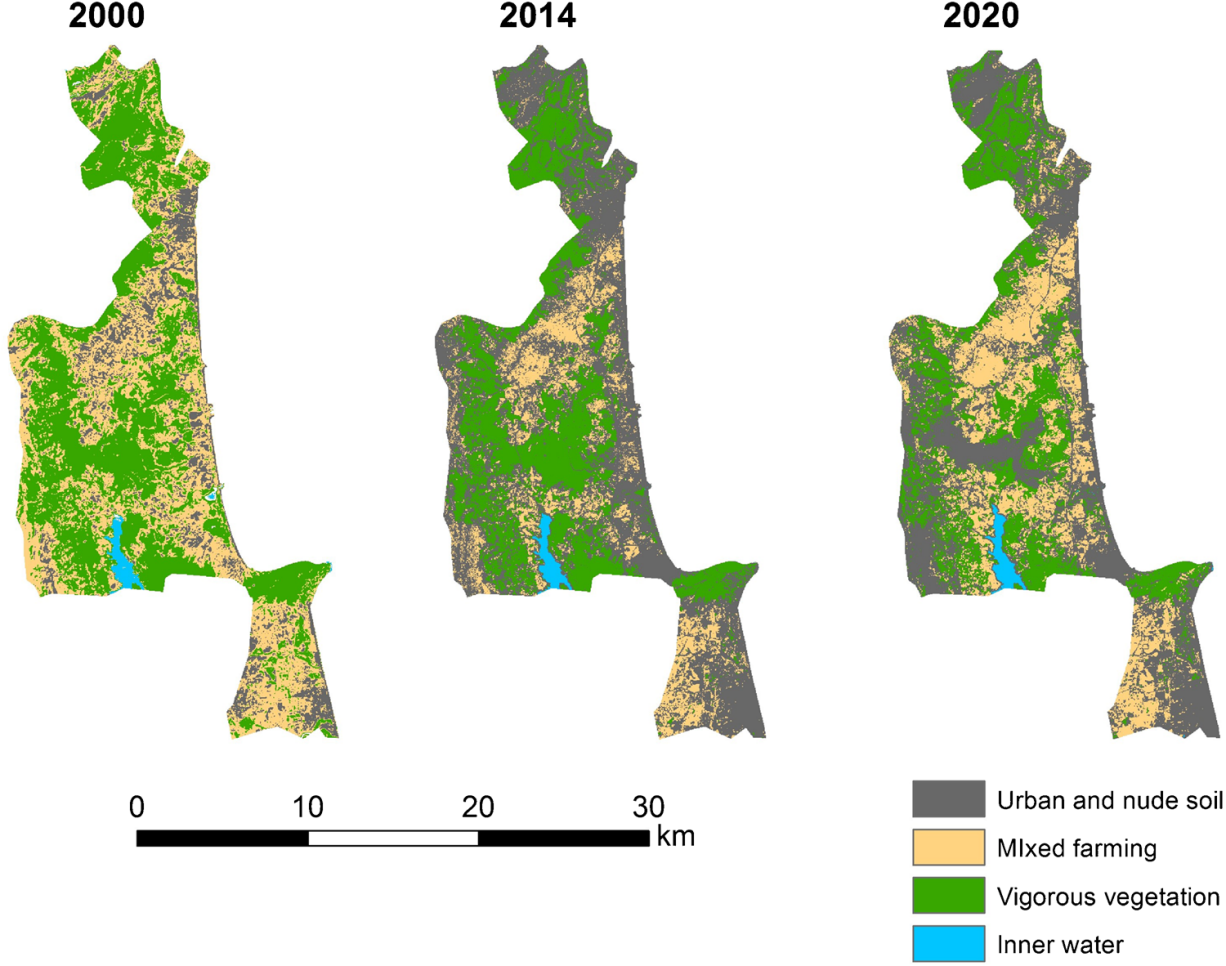
Land use classification in M’diq-Fnideq (prepared by the authors based on USGS)

In August 2020, there is a recovery of vigorous vegetation and mixed farming, with the decline of urban land and bare soil. In the latter case, it has been possible to verify the recovery of extraction and construction areas that are being progressively occupied by vegetation: (Fig. 2) growth of housing construction and (Fig. 3) construction growth. To validate the results, it has been validated in the field. The following figures, Fig. 4., Fig. 5, and Fig. 6, show a sample of recent urbanization (Marina Smir, Mdi’q-Fnideq) and another in rural areas (Halka Zaitoune, Tetouan): evolution of land uses in Halka Zaitoiune, 2006–2020.

### Verification

**Fig. 7:**
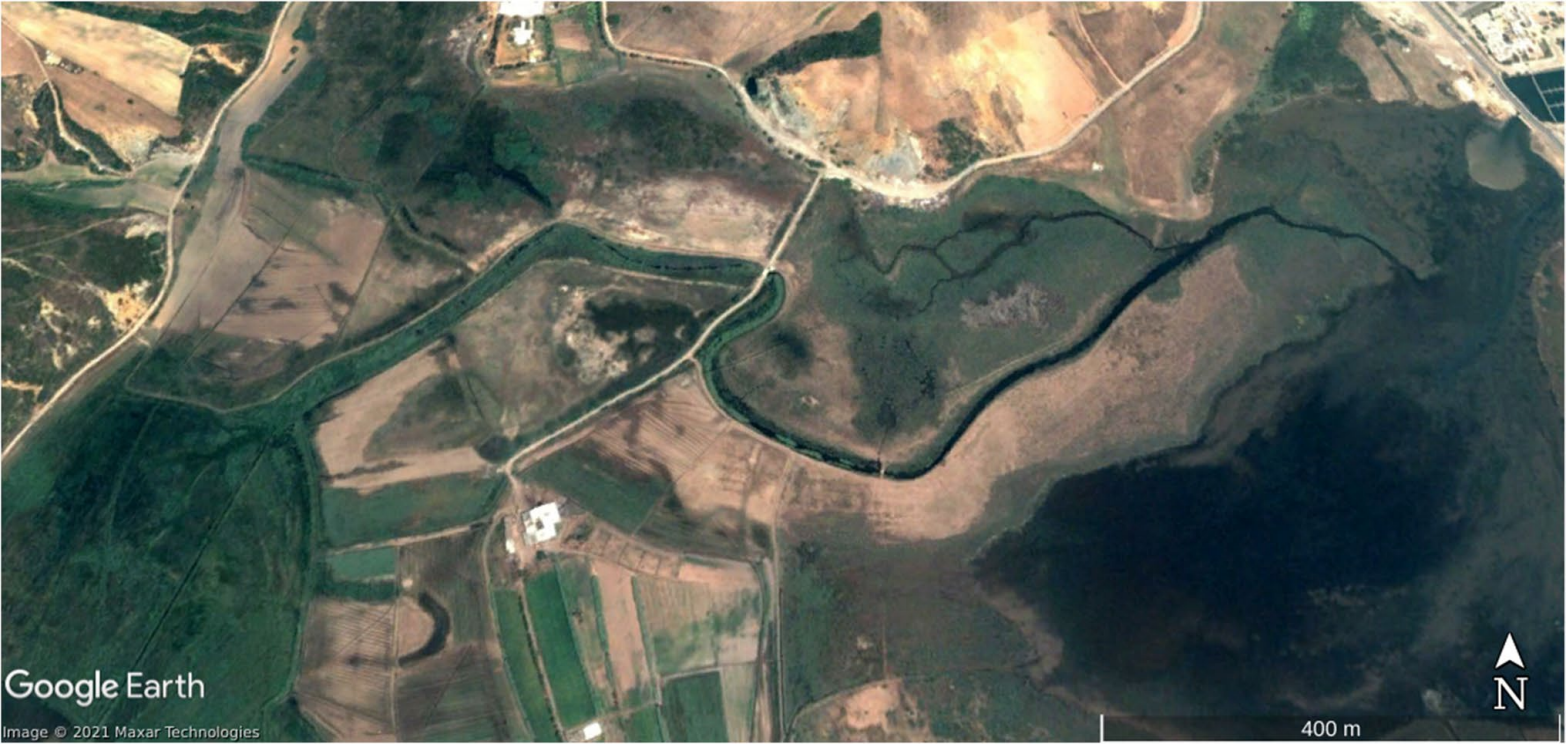
Marina Smir: before development (initial situation, July 2003) (prepared by the authors with Google Earth Pro)

**Fig. 8:**
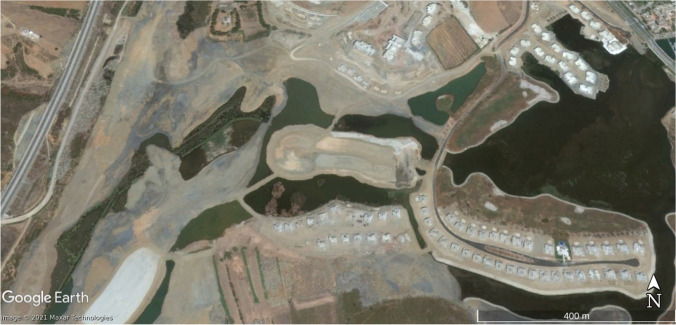
Marina Smir: development in progress (maximum activity, August 2014) (prepared by the authors with Google Earth Pro)

**Fig. 9:**
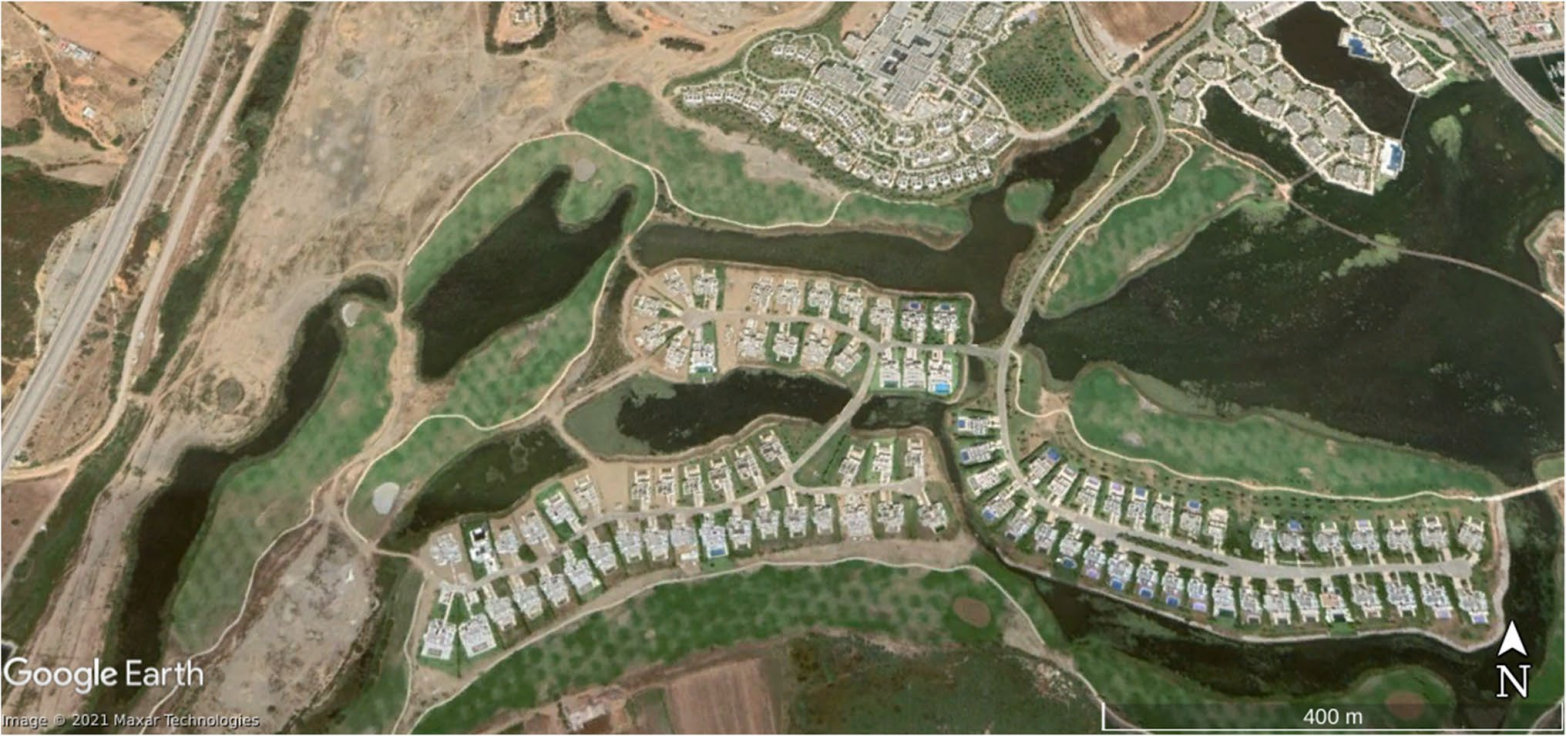
Marina Smir: main works are over, and new chalets are already in use, August 2020 (prepared by the authors with Google Earth Pro)

**Fig. 10:**
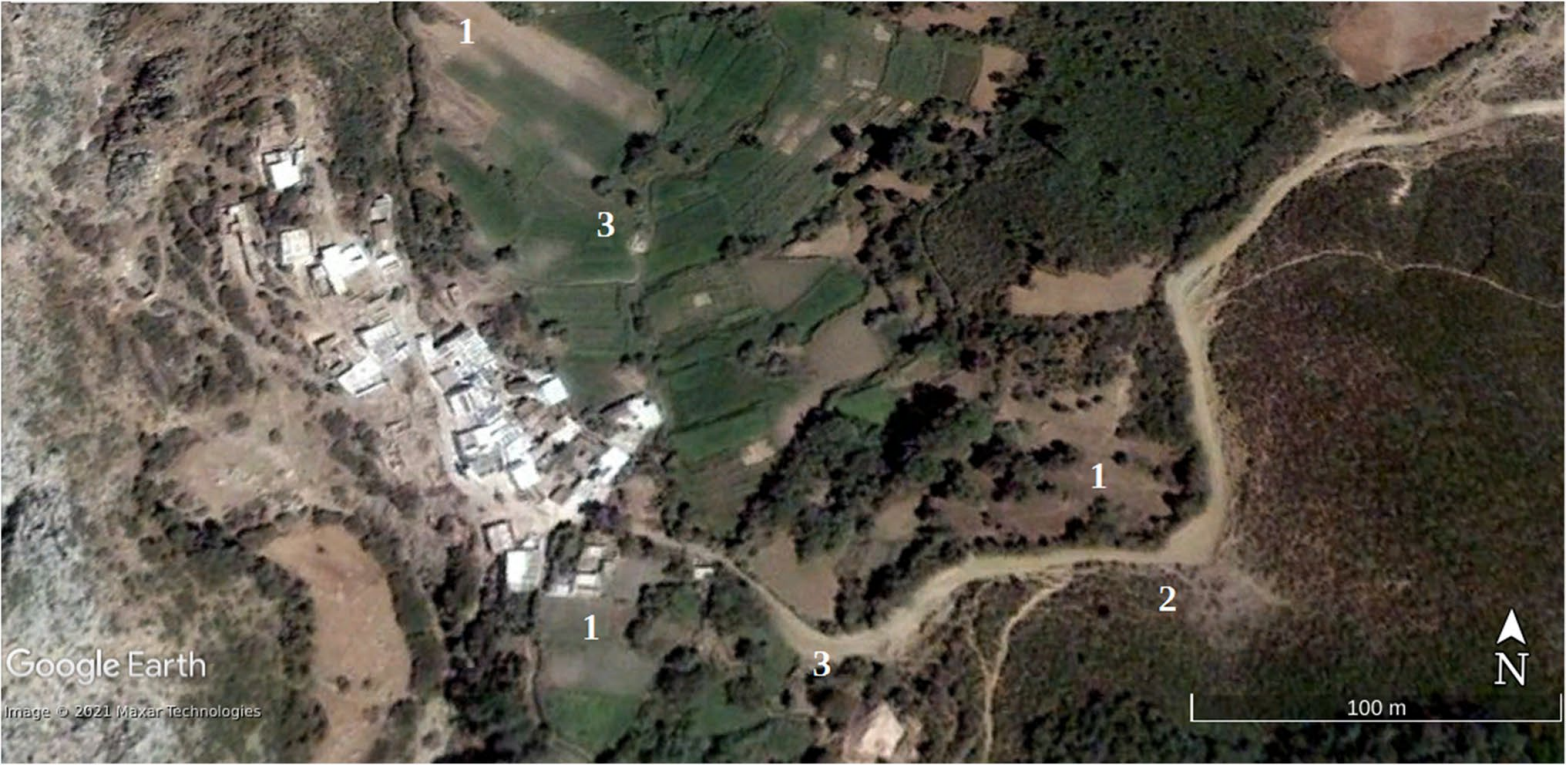
Halka Zaitoune: initial situation, July 2006. (**1**) New farming area added later, including fruit trees in 2020. (2) Fire destruction in 2014. (3) New housing in 2020 (prepared by the authors with Google Earth Pro)

**Fig. 11:**
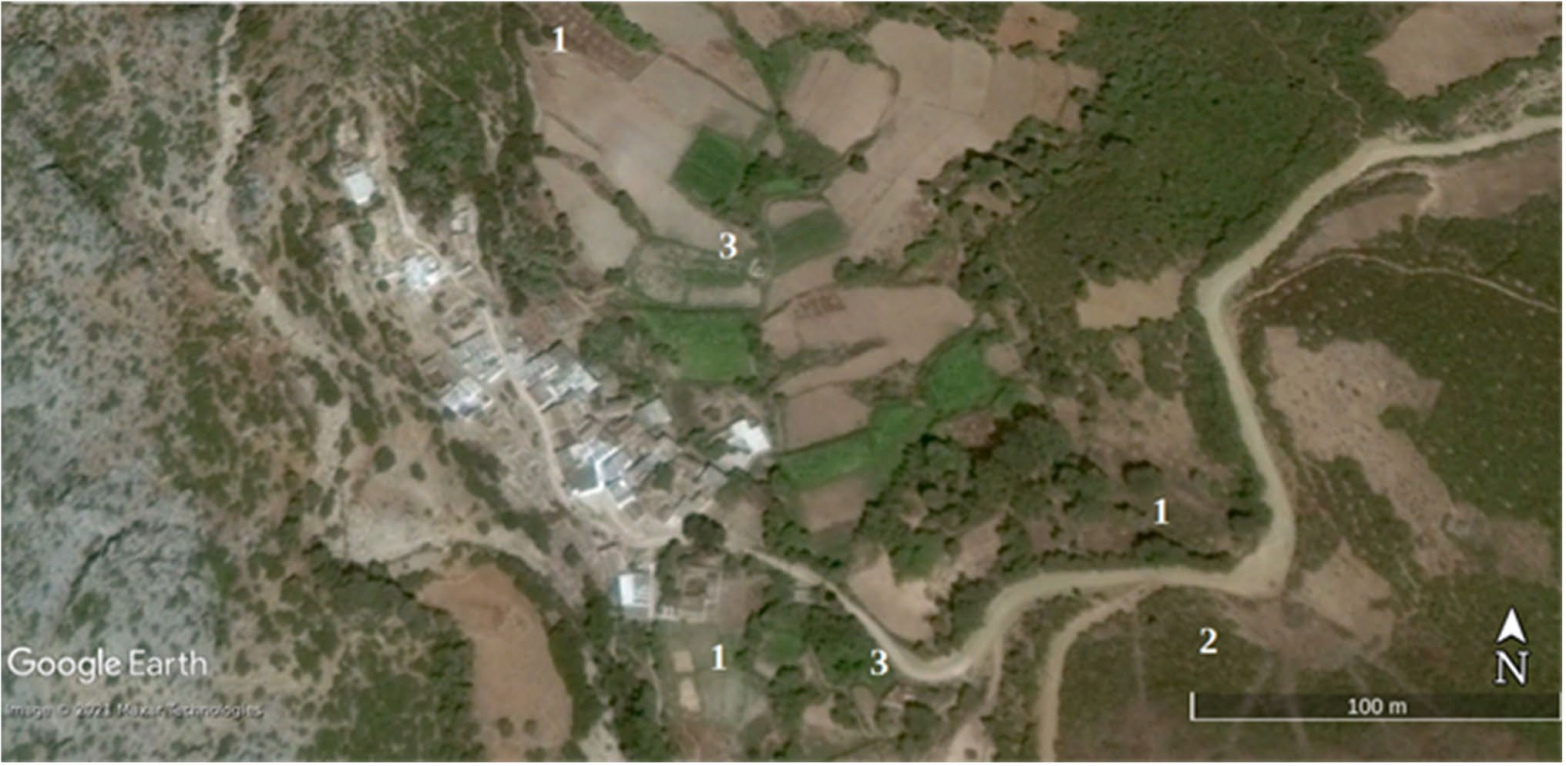
Halka Zaitoune: maximum activities, September 2014. (1) New farming area added later, including fruit trees in 2020. (2) Fire destruction in 2014. (3) New housing in 2020 (prepared by the authors with Google Earth Pro)

**Fig. 12:**
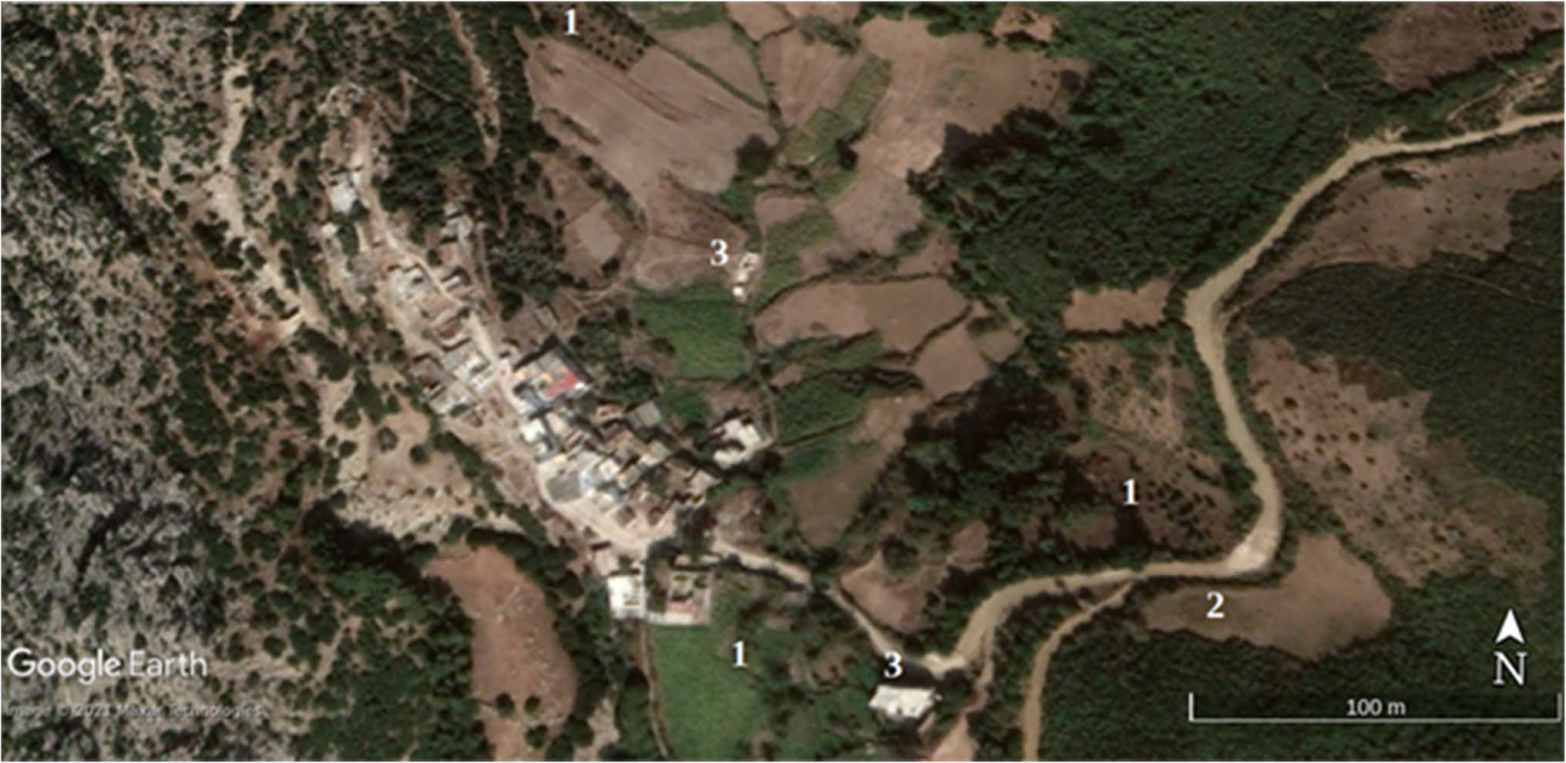
Halka Zaitoune: natural vegetation, September 2020. (1) New farming area added later, including fruit trees in 2020. (2) Fire destruction in 2014. (3) New housing in 2020 (prepared by the authors with Google Earth Pro)

There are many possibilities of choice on the ground to confirm the results obtained from the applied methodology. The cases of Marina Smir (35°42′56.75″ N 5°20′38.94″ W) and Halka Zaitoune (35°31′00.81″N 5°23′57.89″ W) have been taken. In the first case, it is a housing development in a high-standing tourism complex that has been built in an area without prior urbanization (Fig. 7). The construction activities left bare soil during the works, destroying the natural vegetation (Fig. 8). In 2020, the slow recovery of natural vegetation is appreciated, but above all, the vegetation of the new gardens draws attention (Fig 9). The second case is a small rural nucleus with exclusively agricultural activities in July 2006 (Fig. 10). In September 2014, an area devastated by fire appeared with vehicle signs and next to a quarry in September 2014 (Fig. 11). In September 2020, both fire zone and quarry were being colonized again by natural vegetation, while more extension dedicated to cultivation is appreciated with more similarity to the starting situation in 2006 (Fig. 12).

## Discussion

It should be noted that rural settlements in northern Morocco are mainly dispersed. Therefore, a smaller population in rural areas should produce lower values of urban and bare land, as well as a recovery of natural vegetation. This fact has occurred simultaneously with the development of the SDAU which has led to the construction of several HUB macro ports together with a free zone. Part of the agricultural labor force began to be employed in construction and extractive activities. New quarries emerge, and both natural vegetation and cultivation areas give way to the profitable business of extraction and transportation of construction material. The rural exodus has not only emptied the inland municipalities of the population but also reduced the population in dispersed settlements, verifying negative growth in various rural communes. The population is now concentrated in urban and peri-urban areas of the cities of Tangier and Tetouan. Somehow, the evolution of the population and habitat in the province of M’diq-Fnidq does not follow equivalent evolution since the same population increase is not observed. It is one of the areas with the greatest anthropic pressure, with large new residential zones, roads, and tourist facilities. The discrepancy between the data that show the growth of construction and that of the population in the Province of M’diq is due to the fact that it is fundamentally the construction of a second home. In the case of M’diq-Fnideq, the growth of housing exceeds the growth of the population because it is mainly second home constructions. However, it is reasonable to foresee a follow-up of the migratory trend due to the attraction of labor that must be generated by the activities of high-standing tourism that is developing in the area in the medium-term future. This population movement is related to job opportunities generated by the offer, but also to the improvement of communications. This improvement has achieved an increase in mobility between the countryside and the city so that farming areas are losing their inhabitants in favor of the cities. Farmers can move more frequently from their residence in the city to the workplace in the field, evolving toward almost daily mobility. These circumstances have an impact on the number of inhabited houses scattered in the rural habitat, although these buildings remain uninhabited. Land use data show an increase in urban use and bare land between 2000 and 2014, in line with the intense activity in the construction sector, both for infrastructure and for housing. Significant deterioration has also been found due to forest fires, possibly caused by intense activity that is not related to farming. This evolution is reversed toward the year 2020 with an increase in vigorous vegetation and mixed farming in the area of the former provinces of Tangier and Tetouan. The recovery of mixed agricultural areas makes sense considering the recovery of the agricultural workforce, who can now go to work more easily, as the new infrastructure has also spurred mobility. Abandoned quarries give way to natural vegetation again. In the case of Mdiq, it should be noted that a recent forest fire has altered the figures in favor of bare soil. Anyway, it is pointed out as one of the areas with the highest urban pressure (Satour et al. 2021). The role of COVID 19 lock-down has also played an important role in the recovery of natural vegetation, but the August 2020 images in the dry season have been taken only 5 months after; this period is insufficient for the resilience of Mediterranean vegetation with a pronounced dry season (Acácio and Holmgren 2014). This pandemic situation has caused changes in the supply habits of the population due to the reduction in trade flows. It has been found that the population has preferentially resorted to local food supplies in proximity markets related to indigenous agricultural production (Haraoui and Chalabi 2021). We cannot assert that this behavior will stabilize in the medium-term future, but it may be one more reason to suppose a stabilization in the movements of inhabitants between the countryside and the city.

## Conclusion

Even in the absence of updated population data, we can see that migratory pressure has slowed down. We cannot conclude that it has reached a state of saturation but a state of maturity in the vertiginous territorial transformation of the region. Mixed farming has regained its presence, suggesting a return of farmers to their previous labors. Vegetation is recovering much of the lost ground, too, in the absence of extracting activities as much construction activity is over. Population mobility is preferably between home and farms rather than living in the country field. We can also consider an almost daily population movement, but not a displacement of inhabitants as occurs in rural exodus. There are new parks, gardens, pools, and sports areas that affect the classification of land use. The results analyzed together with their verification may indicate a turning point in terms of urban development and population mobility. It is necessary to wait after the current pandemic situation is over to determine the definitive effects on population movement patterns. However, this possible truce may be the right time to heed the consequences of such dramatic and rapid changes. Lack of attention in assessing these transformations is incompatible with reasonable planning for sustainable development.
